# Gingival depigmentation by conservative injectable vitamin C in comparison with scalpel surgery: a randomized controlled split-mouth clinical trial

**DOI:** 10.1186/s13104-025-07476-w

**Published:** 2025-09-30

**Authors:** Manal M. Al-Hajri, Abdulaziz M. Tawfik, Redhwan Saleh Al-Gabri, Sadam Ahmed Elayah, Ahmed Yaseen Alqutaibi

**Affiliations:** 1 Oral Medicine, Oral Diagnosis, Periodontology & Oral Radiology Department, Faculty of Dentistry, Sanàa University, Sanàa, Yemen; 2 Department of Prosthodontics, Faculty of Dentistry, Ibb University, Ibb, Yemen; 3 Oral and Maxillofacial Surgery Department, Faculty of Dentistry, Jiblah University for Medical and Health Sciences, Ibb, Yemen; 4 Substitutive Dental Science Department, College of Dentistry, Taibah University, Al-Madinah, Saudi Arabia

**Keywords:** Gingival pigmentation, Gingival depigmentation, Vitamin c, Scalpel surgery

## Abstract

**Background:**

Gingival pigmentation (GP) is a common aesthetic concern in dentistry. While GP is not harmful, it can cause psychological distress. This study aimed to evaluate the efficacy of vitamin C injection for managing GP compared to the standard scalpel surgery.

**Methods:**

The study involved 20 healthy patients aged 19–36. A randomized split-mouth design was used, with vitamin C injections (group 1) on one side and scalpel surgery (group 2) on the other side of the maxilla. Clinical evaluations of gingival depigmentation were conducted preoperatively and at first, second, three weeks and six months postoperatively, using the Gingival Pigmentation Index (GPI), Melanin Pigmentation Index (MPI), Dummett Oral Pigmentation Index (DOPI) and Color Intensity Analysis using ImageJ. Pain and patient satisfaction were measured with a visual analogue scale (VAS) after treatment.

**Results:**

The GPI, MPI, and DOPI showed no significant differences in pigmentation between the groups at baseline and after six months (*P* = 1.0 and 0.80 for GPI; *P* = 1.00 and 0.54 for MPI; *P* = 1.00 and 0.59 for DOPI). However, after three weeks, greater depigmentation was noted on the surgical side. The vitamin C group reported lower pain and itching levels (itch: *P* = 0.02; pain: *P* = 0.00).

**Trial registration:**

This randomized controlled trial was retrospectively registered in the Thai Clinical Trial Registry at 05-7-2024 (www.thaiclinicaltrials.org), and its registration number is TCTR20240715003.

## Background

The aesthetics of a smile are significantly influenced by the arrangement, morphology, and color characteristics of both the teeth and gingival tissue. The appearance and health of the gingiva are particularly crucial in creating an appealing smile. Generally, the gingiva presents a shade of pink, although this color may vary among individuals due to differences in skin pigmentation. Factors such as the degree of keratinization of the epithelium, the presence of pigment-containing cells, the vascular supply, and the distance to the surface all contribute to variations in gingival color [1]. The presence of gingival melanin pigmentation is often considered a significant aesthetic concern, particularly in cases of excessive gingival display or a “gummy smile” [2].

Gingival hyperpigmentation is characterized by darker gingival tissue beyond the normal range [3]. This condition arises from various substances involved in physiological processes, such as melanin, carotene, and hemoglobin [4], along with contributions from various pathological conditions [5]. Environmental factors like tobacco smoke also play a role in both active and passive forms [6].

Several pathological causes of gingival pigmentation include endocrine disorders (e.g., Addison’s disease, Acromegaly), exposure to heavy metals (e.g., lead, mercury), malignant neoplasms (e.g., Kaposi’s Sarcoma), drug-induced pigmentation (e.g., from minocycline, zidovudine), mucosal conditions (e.g., lichen planus, hemoglobin disorders), tobacco-related pigmentation (smoker’s melanosis), idiopathic conditions (e.g., Peutz-Jegher’s syndrome), and various forms of tattoos (e.g., amalgam, graphite) [7].

Gingival hyperpigmentation is often a genetically inherited trait in certain populations, known as racial or physiological gingival pigmentation. Notably, its prevalence does not show gender disparities. Individuals with darker skin tones are more likely to have pigmented gingiva, while those with fairer skin are less likely [8]. Melanocytes, responsible for producing melanin, can be activated by factors such as sunlight, stress, and hormones. Upon activation, they generate melanosomes through the conversion of tyrosine to dihydroxyphenylalanine (DOPA), catalyzed by the enzyme tyrosinase. This process ultimately leads to the production of either pheomelanin or eumelanin [9].

Evaluation of gingival pigmentation usually relies on clinical indexes such as DOPI, GPI, MPI, and de Krom categories [10–12]. However, these subjective methods can vary due to human factors, resulting in inconsistencies in classifications [13]. To address this, more objective approaches have been investigated, such as visual assessments with color tabs, electronic color measurement devices, and digital pigmentation quantification. However, color tabs can be influenced by observer factors like color blindness and lighting conditions, and optimal gingival shade guides are currently lacking [14]. Quantifying gingival pigmentation with spectrophotometers or colorimeters has been reported [15, 16], but these methods also show limited reproducibility and high variability across different systems [17].

Several surgical techniques are employed for gingival depigmentation, including scalpel surgery [18], electrosurgery [19], cryosurgery [20], laser treatment [21], bur abrasion [22], and radiosurgery [23]. Chemical agents, like a mixture of 90% phenol with 95% alcohol [24], and ascorbic acid (vitamin C) [25, 26], are also used. Additionally, other methods such as masking pigmentation with a free gingival graft [27], or using an acellular dermal matrix allograft [28] are explored.

The scalpel surgical technique, or split-thickness epithelial excision, removes gingival epithelium with a scalpel, allowing connective tissue to heal by secondary intention and resulting in a lighter, melanin-free appearance [20, 29]. This method is straightforward, cost-effective, and efficient, promoting faster healing than other surgical techniques [4, 30]. While it has lower costs and recurrence rates, it may cause pain, post-operative discomfort, and bleeding, requiring a periodontal dressing [30]. This technique is also unsuitable for patients with thinner gingival biotypes or narrow papillary regions [31].

Recent literature indicates no significant differences in depigmentation results or wound healing between surgical scalpel depigmentation and diode laser methods for managing physiological gingival melanosis. Although diode lasers lead to less postoperative pain and bleeding, the scalpel technique demonstrates better healing for depigmented wounds. Both methods are equally effective in promoting wound healing, and repigmentation can occur with either technique [32, 33].

Vitamin C has a notable impact on melanocytes, affecting their function and the interaction between melanocytes and keratinocytes. Disruption in this interaction can influence melanin production. Vitamin C binds to melanin, leading to its fading, and higher doses can result in gingival depigmentation [34]. By inhibiting tyrosinase activity, vitamin C reduces dopaquinone formation, thereby decreasing melanin synthesis [25]. When managing gingival hyperpigmentation, vitamin C injections have shown earlier and more favorable outcomes than topical gels [35]. This non-surgical method is effective, particularly in patients with a thin gingival biotype [36, 37], and is both cost-effective and minimally invasive [38]. Oral mesotherapy with injectable vitamin C has been found to be as effective as scalpel surgery in reducing gingival hyperpigmentation [39].

Most studies on gingival melanin depigmentation are limited to case reports [40, 41], case series [36, 42–44], one non-randomized controlled trial [37], and a few parallel-group randomized controlled trials (RCTs) [39, 45–47] which typically have short follow-up periods of around three months [39, 45, 46], with one study reporting a follow-up of six months [47]. There is a scarcity of studies directly comparing the efficacy of vitamin C injections to surgical techniques, and none that employ a split-mouth RCT for this comparison. Given these limitations, there is a need for a split-mouth RCT with a six-month follow-up to evaluate and compare the effectiveness of intragingival vitamin C injections against scalpel surgery. This study aimed to fill this research gap by assessing the efficacy of vitamin C injections in managing gingival pigmentation using a split-mouth RCT, hypothesizing that these injections offer comparable effectiveness to scalpel surgery.

## Materials and methods

### Calculation of the sample size

The sample size was determined using G*power v3.1.9.2 based on the effect size from Chhina et al. [18]. Parameters were set to α = 0.05, power = 0.95, and an allocation ratio of 1. This calculation indicated a required sample size of 18 patients (36 sides). To account for potential dropouts, the sample size was increased by 10% (2 patients), resulting in a final sample size of 20 patients (40 sides).

### Ethical approval

The study was approved by the ethics committee of Sanaa University (Approval No. 2022/477) and adhered to the Helsinki Declaration (1975, revised 2013). We conducted a split-mouth clinical trial to compare the effectiveness of vitamin C injections and scalpel surgery for gingival depigmentation, registered with the Thai Clinical Trial Registry (TCTR20240715003). All participants were informed and consented prior to participation.

### Recruitment of patients and inclusion/exclusion criteria

From March 2022 to July 2023, 20 patients seeking treatment for melanin pigmentation in the aesthetic region were assessed for eligibility at the Faculty of Dentistry, Sanaa University. Inclusion criteria were physiological gingival pigmentation in the aesthetic region, aesthetic concerns, ages 18 to 50, and good overall health. Exclusion criteria included a thin gingival phenotype, periodontitis, history of drug use causing pigmentation, smoking, pregnancy or lactation, and inability to attend follow-up visits.

### Randomization

**Fig. 1:**
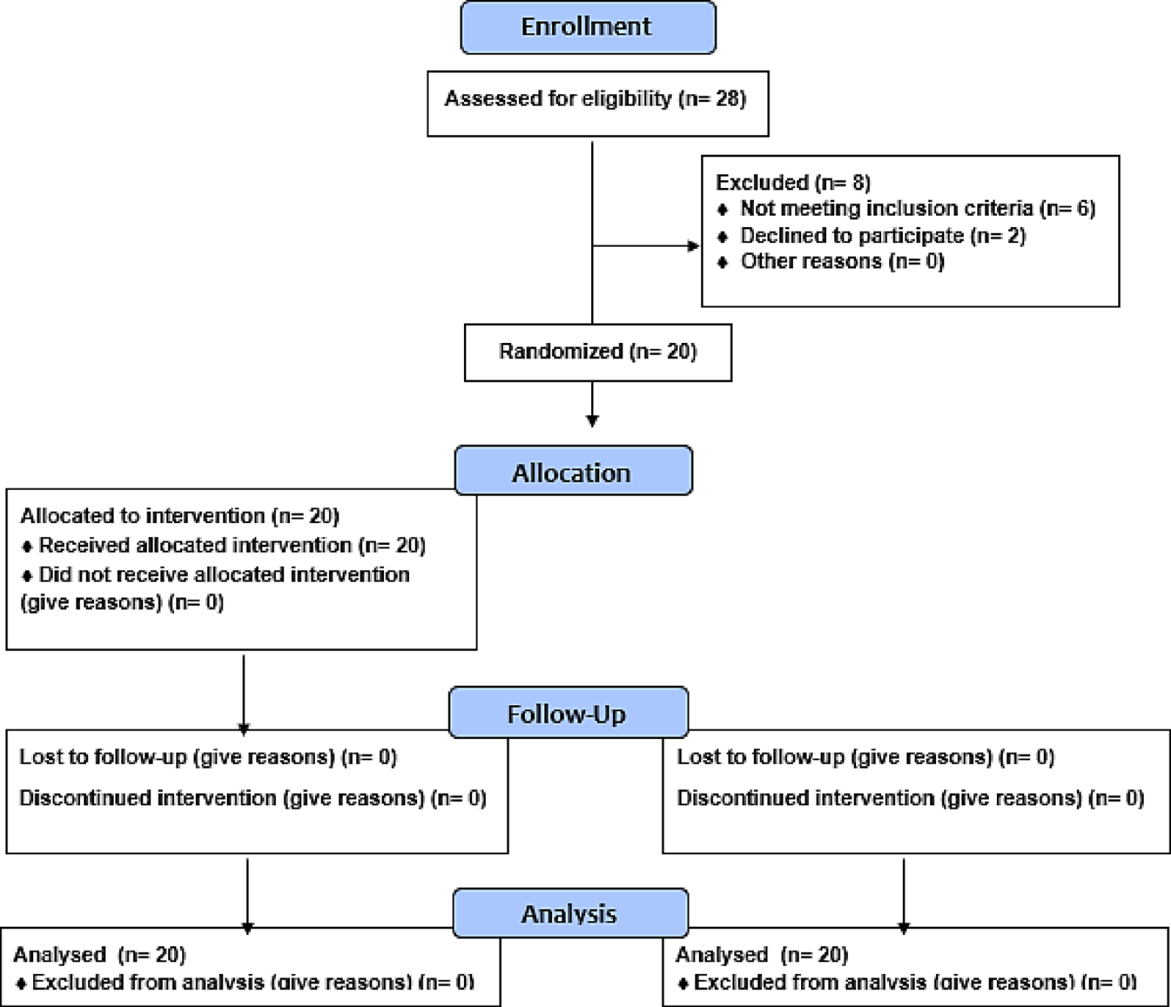
The CONSORT flow diagram

Eligible patients were randomly assigned using a computer-generated system (https://www.randomizer.org/) to receive one of two interventions on either side of the maxilla. Group 1 received intragingival vitamin C injections for depigmentation, while Group 2 underwent surgical depigmentation using a scalpel. Each patient received one treatment on one side and the other on the opposite side, with opaque envelopes sealed by an independent investigator indicating the intervention. Blinding the surgeon was not possible. Our study adheres to CONSORT guidelines and is reported according to the Extension to the CONSORT 2010 checklist for within-person randomized trials (Fig. 1).

### Preoperative preparation phase

Depigmentation protocols included professional scaling using ultrasonic scalers and comprehensive oral hygiene instructions to ensure optimal oral health conditions before any procedure.

### Preoperative clinical measurements

 At the start, all patients underwent preoperative clinical measurements, including the plaque index (PI) [48] and gingival index (GI) [49] to assess gingival health. The GPI [24] was used with scoring criteria ranging from 0 (no pigmentation) to 3 (diffuse pigmentation). The MPI assessed pigmentation distribution, scoring from 0 (no pigmentation) to 2 (continuous ribbon of pigmentation) [50]. The DOPI evaluated pigmentation intensity, scoring from 0 (no pigmentation) to 3 (heavy pigmentation) [10].

### Study procedures

#### Vitamin C injection procedure

**Fig. 2:**
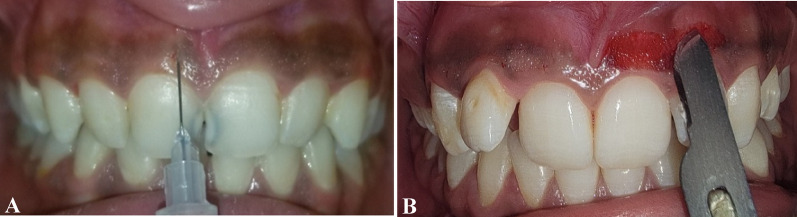
**A** Intra-epithelial injection of vitamin C. **B** Gingival depigmentation by scalpel surgery

**Fig. 3:**
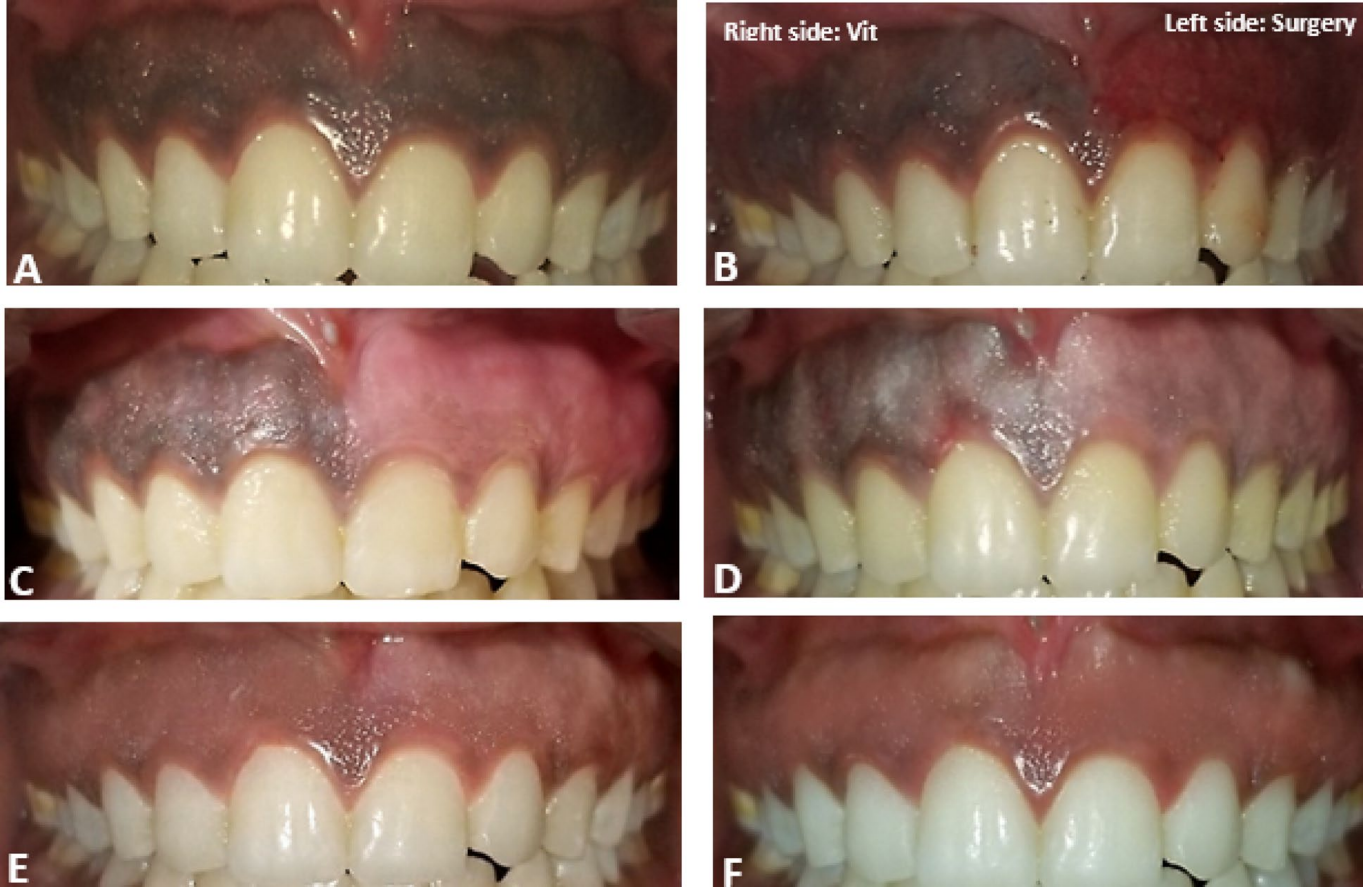
Case presentation images: **A** Preoperative image shows heavy brown to black pigmentation on both sides. **B** Immediate postoperative image after Vitamin C injection (right side) and scalpel surgery (left side). **C**,**D** and **E** One-week, two-weeks, and three-weeks follow-up images, respectively, reveal vitamin C side shows fainting of gingival pigmentation and small areas of pink color begin to occur, more fainting and a whitish coat cover the pigmented area and further fainting of pigmentation and absence of black pigmentation and pink color becomes more spread. **F** After six months, stabilization of pink color without recurrence occurs on both sites

**Fig. 4:**
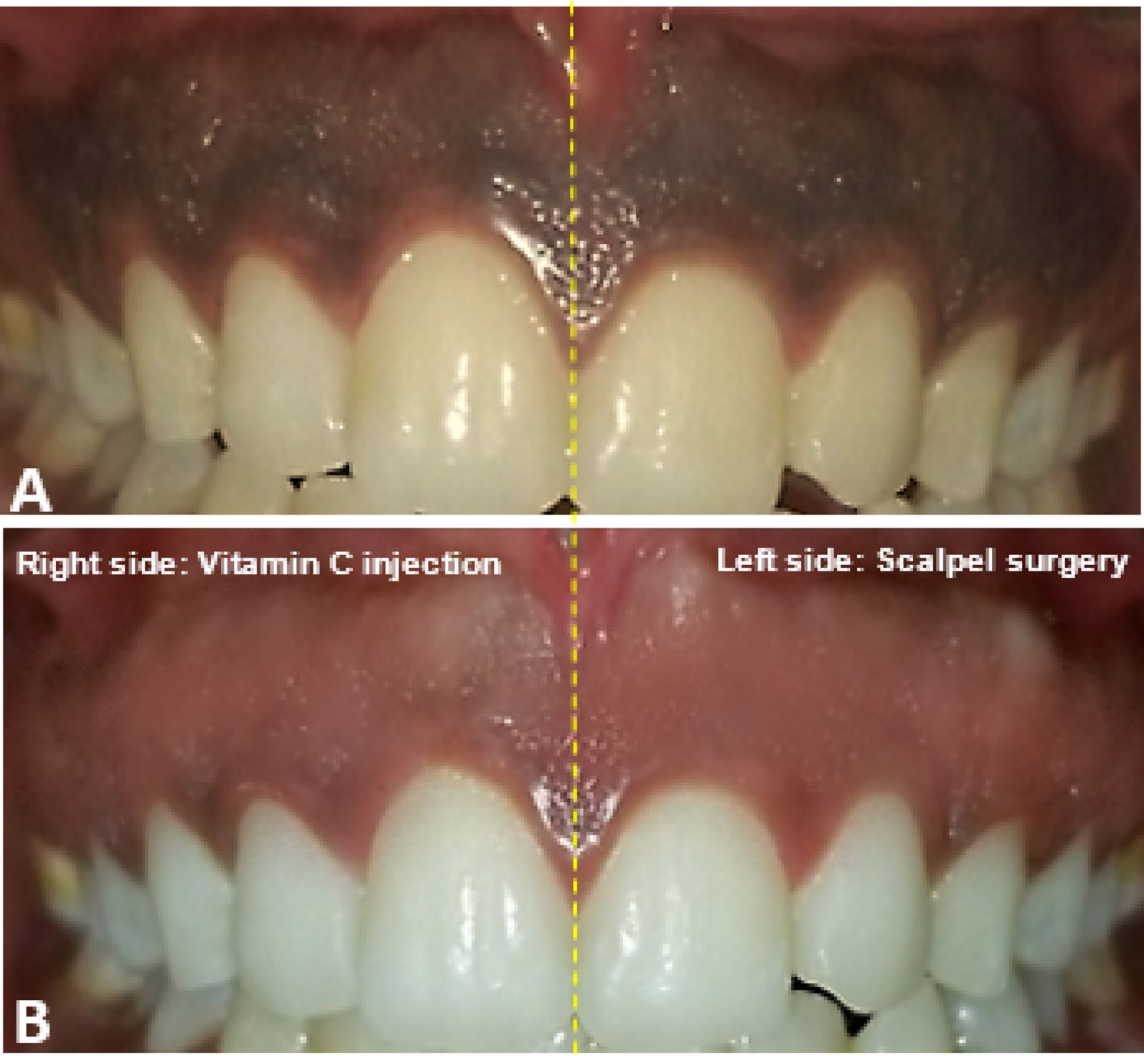
**A** Preoperative image shows heavy brown to black pigmentation on both sides. **B** Six months postoperative

 The site was anesthetized using 2% lidocaine with adrenaline 1:80.000. An intragingival injection of vitamin C at a concentration of 250–375 mg (Shandong Xinhua Pharmaceutical Co.) was administered using a 30-gauge syringe. The needle was bent at 45° with a bevel of the needle oriented upward and inserted parallel to the superficial gingival tissue. The needle was inserted to a depth of 0.5 mm to 1.0 mm at intervals of 2 mm to 3 mm (Fig. 2A). A volume of 0.1 mL of vitamin C was administered at each point until blanching of the gingiva was noted, repeated weekly for three weeks [37, 47] (Figs. 3 and 4). Post-operative instructions were provided, advising the patient to avoid citrus fruits, spicy foods, and foods containing color additives.

### Scalpel surgery procedure

 Local anesthesia was administered via infiltration using 2% lidocaine with adrenaline (1:80,000) at the surgical site. The pigmented epithelial layer and a thin connective tissue layer (partial thickness incision) were scraped with a No. 15 scalpel blade. Surgical depigmentation began at the midline and progressed laterally, ensuring the blade remained parallel to the tooth axis to avoid severing the junctional epithelium or exposing the underlying bone. Subsequently, the exposed connective tissue surface was carefully examined, and the wound was irrigated with saline (Fig. 2B). The depigmented area was covered with a periodontal dressing (Maquira Pericem Perio Pak 180 g, Brazil) after controlling bleeding with sterile gauze and direct pressure, and it was dressed for one week. Patients were advised on oral hygiene but instructed to avoid brushing the treated area for one week. A regimen of antibiotics (Amoxicillin 500 mg), analgesics (Ibuprofen 200 mg), and saline rinses was prescribed (Figs. 3 and 4)

### Outcomes evaluation

**Fig. 5:**
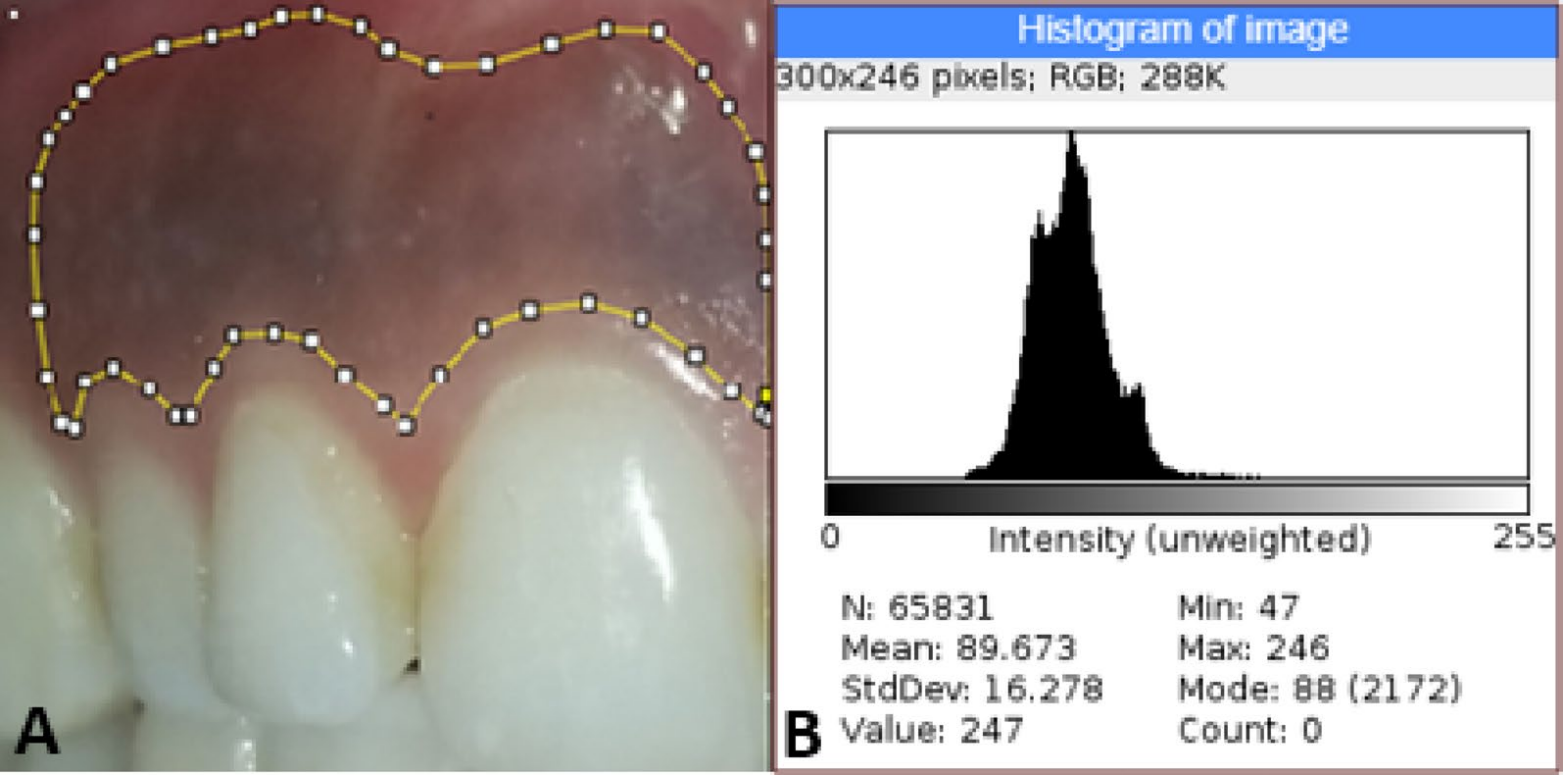
Preoperative photograph bordered (**A**) and analysis the pigmented area using color assessment software (ImageJ) (**B**)

**Fig. 6:**
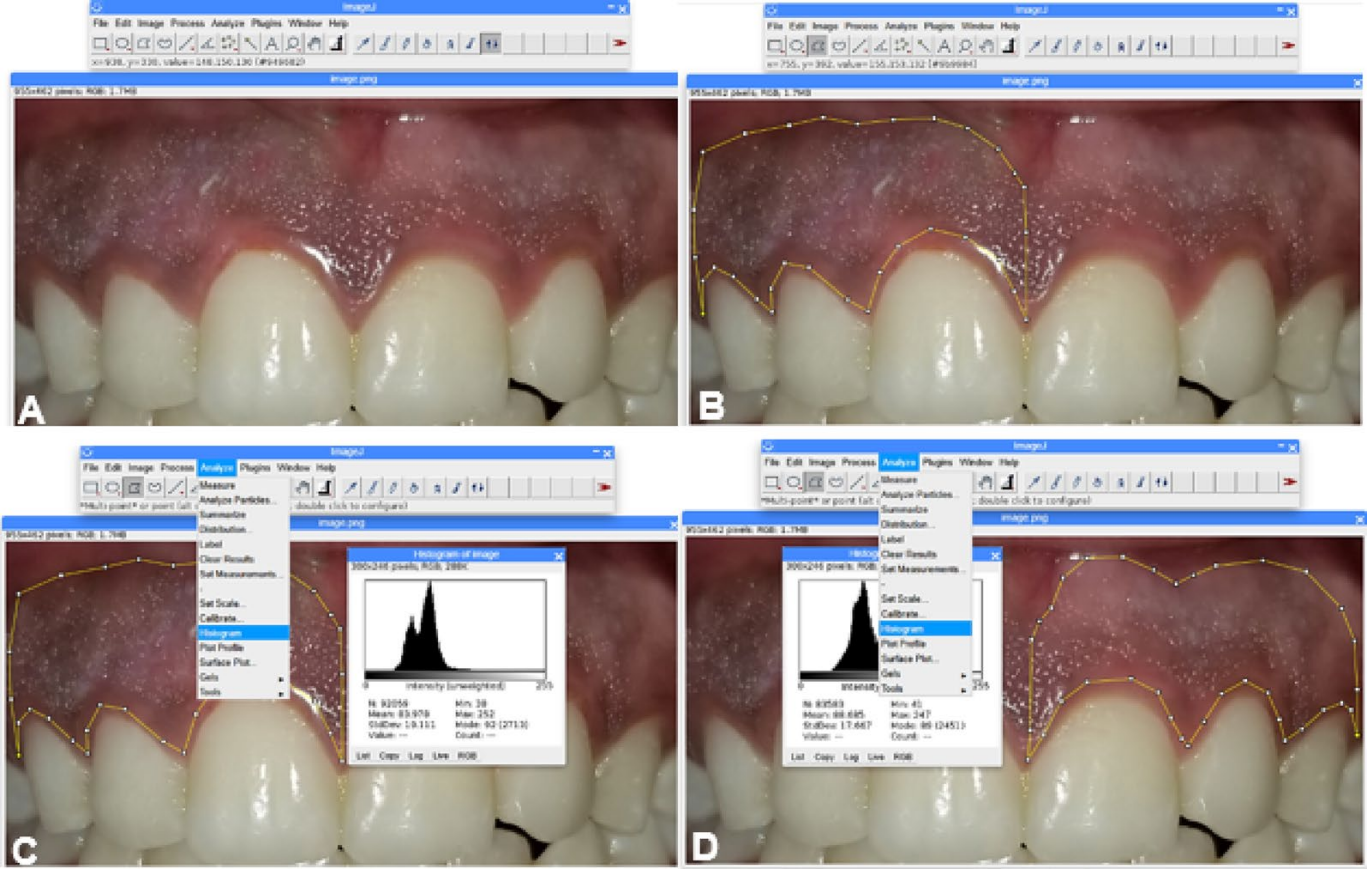
Analysing the intensity values using ImageJ software: **A**, The gingiva from the left and right distal-cervical regions of the maxillary canine was cropped using Adobe Photoshop. **B**, The gingiva from the right distal-cervical region of the maxillary canine to the midline for the vitamin C side was outlined with the polygonal lasso tool. **C** and **D**, Color intensity was assessed with the histogram feature, calculating the mean and standard deviation of the intensity values for vitamin C and surgical side respectively

 Gingival pigmentation was assessed using gingival and melanin indices at baseline, three-weeks post-procedure, and six months post-operatively. Standardized digital photographs were captured using a 12MP camera and analyzed for color intensity with ImageJ software [51]. Photographs were taken in the morning under daylight in the same dental unit at baseline, and after one, two, three weeks, and six months. The camera was positioned 90 mm from the mouth during these sessions. The gingiva from the left and right distal-cervical regions of the maxillary canine for both groups sides was cropped using Adobe Photoshop. The cropped image was imported into ImageJ software to analyze the intensity values. The distance between these two points was standardized to 10 cm. The gingiva from the right distal-cervical region of the maxillary canine to the midline for vitamin C side was bordered by polygonal selection using polygonal lasso tool. Color intensity was assessed using the histogram feature and the mean and standard deviation of the intensity values were calculated. The same procedures were applied for left side (surgical side) as shown in Figs. 5 and 6.

Patient pain and itching were evaluated using the VAS, divided into vertical (itching) and horizontal (pain) Sections [52]. Patient satisfaction was assessed six months later using a five-grade self-assessment scale [18, 53].

### Statistical analysis

 Data was collected, computed, and analyzed using SPSS version 20.0. Color intensity values showed a normal distribution. Group comparisons were conducted using independent sample t-tests (*p* < 0.05), while within-group analysis used paired sample t-tests (*p* < 0.05). The Marginal Homogeneity Test analyzed categorical variables.

## Results

No adverse events were observed after consent and enrollment. Participants’ ages ranged from 19 to 36 years, with an average of 27.5 years. Repigmentation occurred in 6 out of 20 patients (30%) during the six-month follow-up, with higher recurrence noted in cases of heavy pigmentation, particularly on the surgical side.

There were no significant differences in pigmentation between the scalpel surgery and vitamin C injection groups at baseline and after six months, based on GPI, MPI, and DOPI results (*P* = 1.0 and 0.80 for GPI; *P* = 1.00 and 0.54 for MPI; *P* = 1.00 and 0.59 for DOPI). However, significant differences were found after one, two, and three weeks (*P* = 0.00, 0.00, and 0.01 for GPI; *P* = 0.00, 0.00 and 0.001 for MPI; *P* = 0.000, 0.000 and 0.002 for DOPI), with greater depigmentation observed on the surgical side, as shown in Tables 1 and 2, and 3.

**Table 1:** Comparison of the pigmentation at baseline, after one, two, three weeks, and six months later between the two groups using GPI

Time	Treatment Type	GPI								Mean	Std. D.	*P*- value
		Absence		Spots		Patch		Diffuse				
Baseline	Surgery	0	0%	0	0%	7	35%	13	65%	2.65	± 0.48	1.0
	Vitamin C	0	0%	0	0%	7	35%	13	65%	2.65	± 0.48	
One week	Surgery	17	85%	3	15%	0	0%	0	0%	0.15	± 0.36	0.00*
	Vitamin C	0	0%	5	25%	11	55%	4	20%	1.95	± 0.68	
Two weeks	Surgery	12	60%	8	40%	0	0%	0	0%	0.40	± 0.50	0.00*
	Vitamin C	0	0%	12	60%	8	40%	0	0%	1.40	± 0.50	
Three weeks	Surgery	7	35%	12	60%	1	5%	0	0%	0.70	± 0.57	0.01*
	Vitamin C	0	0%	19	95%	1	5%	0	0%	1.05	± 0.22	
Six months	Surgery	2	10%	14	70%	4	20%	0	0%	1.10	± 0.55	0.80
	Vitamin C	1	5%	14	70%	5	25%	0	0%	1.20	± 0.52	

*Significant at *P* ≤0.05

**Table 2:** Comparison of the pigmentation at baseline, after one, two, three weeks, and six months later between the two groups using MPI

Time	Treatment type	MPI						Mean	Std. D.	*P*- value
		Absence		Solitary		Continuous				
Baseline	Surgery	0	0%	4	20%	16	80%	1.80	± 0.41	1.00
	Vitamin C	0	0%	4	20%	16	80%	1.80	± 0.41	
One week	Surgery	17	85%	3	15%	0	0%	0.15	± 0.36	0.00*
	Vitamin C	0	0%	11	55%	9	45%	1.45	± 0.51	
Two weeks	Surgery	13	65%	7	35%	0	0%	0.35	± 0.48	0.00*
	Vitamin C	0	0%	17	85%	3	15%	1.15	± 0.36	
Three weeks	Surgery	7	35%	13	65%	0	0%	0.65	± 0.48	0.001*
	Vitamin C	1	5%	19	95%	0	0%	0.95	± 0.22	
Six months	Surgery	2	10%	18	90%	0	0%	0.90	± 0.30	0.54
	Vitamin C	1	5%	19	95%	0	0%	0.95	± 0.22	

*Significant at *P* ≤0.05

**Table 3:** Comparison of the intensity of pigmentation at baseline, after one, two, three weeks, and six months later between the two groups using DOPI

Time	Treatment Type	DOPI								Mean	Std. D.	*P*- value
		Absence		Mild		Moderate		Severe				
**Baseline**	**Surgery**	0	%	2	10%	8	40%	10	50%	2.40	± 0.68	1.00
	**Vitamin C**	0	0%	2	10%	8	40%	10	50%	2.40	± 0.68	
**One week**	**Surgery**	17	85%	3	15%	0	0%	0	0%	0.15	± 0.36	0.000*
	**Vitamin C**	0	0%	6	30%	7	35%	7	35%	2.05	± 0.82	
**Two weeks**	**Surgery**	13	65%	7	35%	0	0%	0	0%	0.40	± 0.50	0.000*
	**Vitamin C**	0	0%	7	35%	11	55%	2	10%	1.75	± 0.63	
**Three weeks**	**Surgery**	7	35%	13	65%	0	0%	0	0%	0.65	± 0.48	0.002*
	**Vitamin C**	2	10%	9	45%	9	45%	0	0%	1.35	± 0.67	
**Six months**	**Surgery**	2	10%	16	80%	2	10%	0	0%	1	± 0.45	0.59
	**Vitamin C**	1	5%	15	75%	4	20%	0	0%	1.15	± 0.48	

*Significant at *P* ≤0.05

A significant decrease in mean scores from the initial assessment to subsequent follow-up visits demonstrated the efficacy of vitamin C as a depigmenting agent. In a clinical comparison using GPI and MPI, the baseline mean GPI was 2.65, decreasing to 1.95 after one week and 1.05 after three weeks. Similarly, the baseline mean MPI was 1.80, declining to 1.45 after one week and 0.95 after three weeks, showing a statistically significant difference from baseline to six months.

**Fig. 7:**
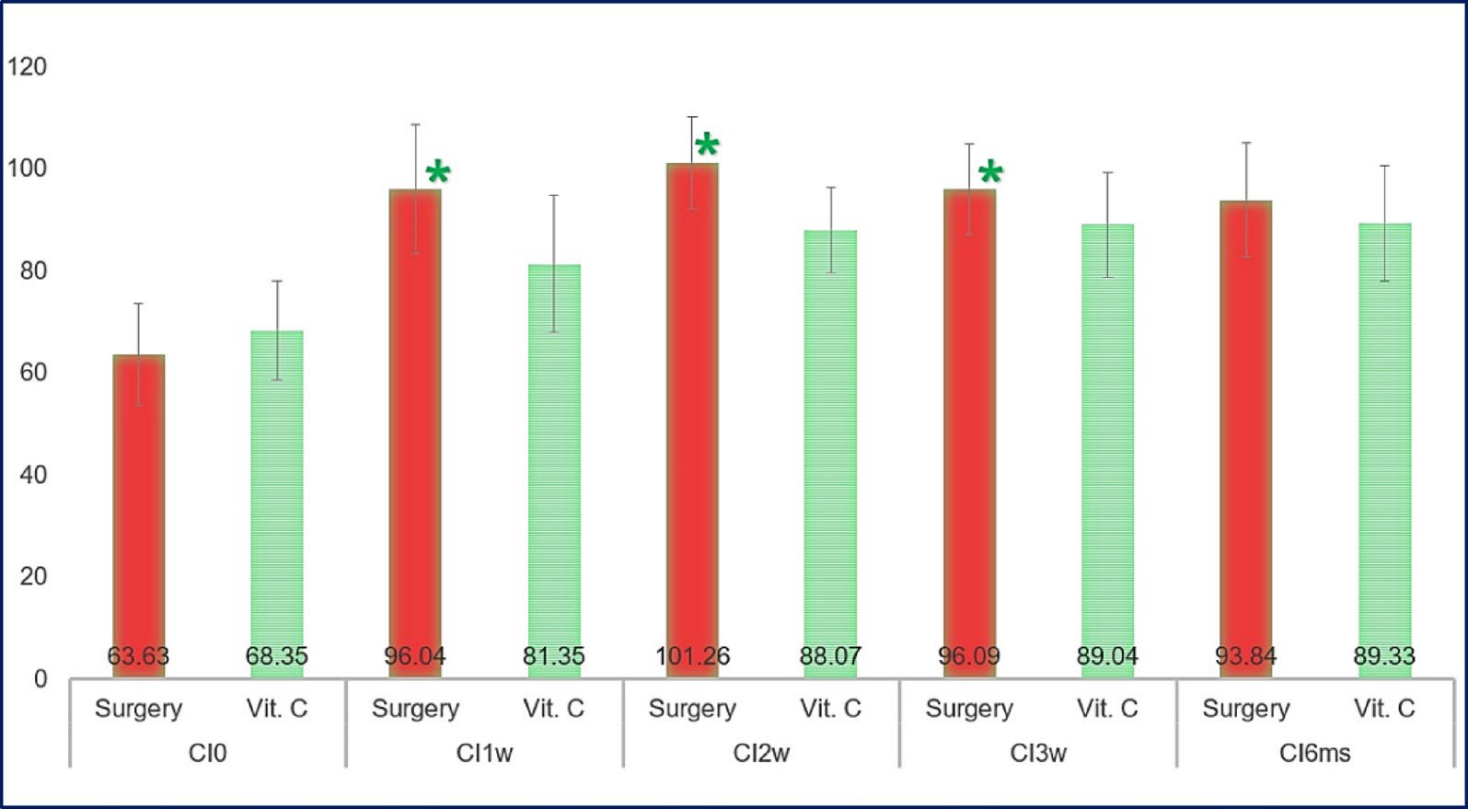
Comparison of the color intensity (CI) between groups (sides)

Color assessment software, specifically ImageJ, revealed no significant differences in color intensity between the two groups at baseline and after six months (*P* = 0.137 and 0.212 respectively). However, statistically significant differences were observed after one, two, and three weeks (*P* = 0.001, 0.000, and 0.026, respectively), with the surgical side showing greater depigmentation, indicating lighter pigmentation (Fig. 7).

The vitamin C injection side exhibited lower levels of pain and itch compared to the scalpel surgery side. After one week, there was a significant difference in itch (*P* = 0.02) and pain (*P* = 0.00) VAS scores between the groups. However, after six months, patient satisfaction did not show a significant difference between the two methods (*P* = 0.08).

## Discussion

Gingival pigmentation is a prevalent concern in terms of aesthetics, prompting individuals to seek treatment despite its non-medical nature. While it poses no health risks, many desire its removal for cosmetic reasons [29]. Various treatments exist, with surgical depigmentation favored for its simplicity, cost-effectiveness, and high success rates [54], but drawbacks include postoperative bleeding, pain, and anxiety due to its invasive nature [20]. In contrast, vitamin C injections offer a minimally invasive alternative that can effectively reduce pigmentation while promoting overall gingival health [37, 44, 47].

Vitamin C is renowned for its antioxidant properties and depigmenting abilities. It has been successfully administered through intradermal, intramucosal, and topical routes [25, 29, 34, 44]. The primary mechanism involves interaction with copper ions at the active site of tyrosinase, inhibiting its function and reducing melanin synthesis. Furthermore, ascorbic acid plays a crucial role in postoperative tissue healing by facilitating collagen synthesis and fibroblast differentiation [55, 56].

 Our study compared the efficacy of intragingival vitamin C injections and scalpel surgery for gingival depigmentation using a split-mouth RCT design with a six-month follow-up, aiming to advance depigmentation techniques and improve patient care. Minimizing inter-individual variability among groups is essential in treatment research, as it can influence baseline conditions and treatment responses. Consequently, a split-mouth study design was employed in this study to eliminate confounding factors like patient’s sex, age, and pigmentation level, allowing for a more precise comparison and improving the internal validity of the findings. Furthermore, the significance of RCTs lies in their ability to reduce bias and enhance the reliability of study outcomes.

 We found no significant differences in pigmentation between the scalpel surgery and vitamin C injection groups at baseline and six months. However, significant depigmentation occurred in the surgical group at one, two, and three weeks. The DOPI showed similar results, with more depigmentation initially on the surgical side. Although the vitamin C group experienced less pain one-week post-treatment, satisfaction levels were similar after six months. Overall, scalpel surgery achieved faster and more pronounced depigmentation, allowing us to reject the null hypothesis that vitamin C injections are equally effective.

Our results align with previous studies. Yussif et al. observed similar improvements in gingival color when comparing scalpel surgery and vitamin C mesotherapy, further supporting the efficacy of both treatments [37]. Sheel et al. found satisfactory aesthetic outcomes using a combination of scalpel surgery and vitamin C, with low pain levels and no repigmentation over a follow-up period [26]. Haque et al. found that gingival depigmentation using locally injected vitamin C is as effective as surgical methods and offers higher patient satisfaction compared to surgical depigmentation [47]

 At the six-month follow-up, a higher rate of recurrence was observed in cases with heavy pigmentation, particularly on the surgical side. This finding in contrast to previous studies by Yussif et al. [34] and Yussif et al. [37]. which reported a reduction in mean values or an increase in depigmentation on the vitamin C side between the last treatment visit and the follow-up visit, our study found no decrease in mean values or increase in depigmentation on the vitamin C side during this period. This aligns with recent research by Haque et al. who found a repigmentation, as neither of the techniques can permanently eliminate the melanin production ability of the gingival epithelium [47]. 

 ImageJ analysis showed a significant difference in mean values for the vitamin C side during injection visits, with increased gingival lightness. However, no significant difference was found between the last visit and follow-up visits. This aligns with Shamida et al. who noted a greater lightness increase after four weeks of ascorbic acid gel but no significant difference between 8 and 12 weeks [25]. In our study, ImageJ revealed no significant difference between the surgical and vitamin C sides after six months.

Regarding pain and itch, the vitamin C injection group reported lower pain and itch levels than the scalpel surgery group, consistent with previous studies [34, 37, 44]. Pain and itch at the injection site are mainly due to the acidity of vitamin C and the needle prick. The acidity helps achieve optimal tissue penetration. In contrast, pain in the surgical area arises from exposed nerve endings, influenced by penetration depth and the patient’s pain threshold [37]. Patient satisfaction was assessed using the VAS for perceived aesthetic improvement. After six months, no significant difference was found, with mean scores of 3.85 for the scalpel surgery group and 3.60 for the vitamin C injection group, consistent with Haque et al. [47] and Yussif et al. [44], where most patients expressed satisfaction with aesthetic outcomes from vitamin C injections for gingival depigmentation.

This study offers valuable insights while indicating areas for future research, including long-term pigmentation recurrence after vitamin C treatment and its adjunct use with surgery. Further exploration of refined vitamin C dosage and delivery methods to enhance early efficacy is needed, along with larger studies and longer follow-ups to validate these findings and inform clinical protocols.

The study found that vitamin C injections were less effective than surgery for initial depigmentation, with surgery achieving greater results at three weeks. However, by six months, the differences were no longer significant, indicating comparable long-term outcomes. While vitamin C injections had lower pain and itch levels and were better tolerated, overall effectiveness in terms of patient satisfaction was similar to surgery. These results underscore the need for further research to confirm the long-term efficacy of both methods.

 This study had several limitations, including a small sample size and the exclusion of smokers and individuals with systemic diseases, which further limit its applicability. The focus on anterior gingiva also means that patients with broader smiles were not fully considered. Although the split-mouth design helps control variability, it may introduce bias due to patient awareness of the treatments, and the lack of blinding could affect outcome assessments. Lastly, as a preliminary study with a short duration, it may not adequately reflect long-term adverse effects or outcomes. Future research should aim for larger, more diverse populations, longer follow-ups, and double-blind designs while using objective measures of treatment effectiveness.

## Conclusions

 Within the limitations of this study, it could be concluded that both scalpel surgery and vitamin C injection procedures were found to be safe for treating gingival hyperpigmentation, with no adverse events reported during the study. Furthermore, vitamin C injection was as effective as surgery and provided better tolerability with reduced pain levels than surgical methods, although patient satisfaction ratings were similar for both approaches.

## Data Availability

All data generated or analysed during this study are included in this published article.

## Abbreviations

DOPI: Dummett oral pigmentation index
GP: Gingival pigmentation
GPI: Gingival pigmentation index
MPI: Melanin pigmentation index
RCT: Randomized controlled trial
VAS: Visual analogue scale
